# Supplementary material for: Lower Adherence to Lifestyle Recommendations of the World Cancer Research Fund/American Institute for Cancer Research (2018) Is Associated with Decreased Overall 10-Year Survival in Women with Breast Cancer
Source: Nutrients. 2025 Mar 12;17(6):1001. doi: 10.3390/nu17061001 (PMC11945812; doi:10.3390/nu17061001)
Supplement: Supplementary file 1 [file nutrients-17-01001-s001.zip › nutrients-3479459-supplementary.pdf]

Supplementary Tables

Table S1. Protocol for research telephone calls.

Good morning/afternoon, I would like to speak with Mrs. (patient's name).

In case the patient is alive:
My name is (interviewer's name), I am a researcher at UFSC, and I am participating in a research project on nutrition and breast cancer. I contacted you regarding your participation in this project a few months ago. Do you remember answering questions about your diet, weight, and measurements? Do you also remember being asked if you had blood tests?

Currently, we are collecting additional information over the phone to complement this research. We would like to know if you would be willing to participate by answering a few questions, which will take about 8 minutes.

If yes:

1. **Before starting the questions,** I would like to emphasize that all information will remain confidential (participant identities will only be used with consent). Therefore, I would like to send you a Free and Informed Consent Form for you to sign.
   Would you (ma'am) agree to participate in this stage of the research? Could you provide your phone number (WhatsApp) so we can send you the form link? [If you don't use WhatsApp, ask if you have an email.]
2. After her breast cancer treatment, did Mrs. (patient's name) have a new case of cancer in the same breast or in a different location? [If it was in a different location, ask where.]
3. **[If recurrence occurred]** Do you remember when the second tumor was diagnosed? If you have any document with this information, could you kindly inform us of the date? [Say the participant can wait to retrieve the document; if they are unable to find it, tell them they can check later, and schedule a new call to retrieve this information.]
4. **[If recurrence occurred]** Did you undergo any treatment for this second tumor, such as surgery, chemotherapy, radiotherapy, or hormone therapy? If so, what treatments were performed? [If surgery was performed, also ask what type of surgery it was—for example: partial mastectomy, radical mastectomy (total), or oophorectomy.]
5. **[If recurrence occurred and treatment was performed]** In which hospital/healthcare institution did you undergo this/these treatment(s) for the second tumor?
6. [If you have had and undergone treatment(s)] Do you remember when you started this/these treatment(s) and when you finished?
7. Are you currently undergoing any treatment related to the tumor? If so, what type of treatment are you undergoing?
8. Are you undergoing oncological treatment currently? Did you have any complications, that is, any problems during this treatment that required hospitalization?
9. Do you have any health problems, besides the cancer, that you have had? Which one(s)?
10. Are you currently taking any medication? Which one(s)?

[If the participant has pending information to provide] Then, I will call you again tomorrow to get the information listed in the consent form for the cancer study (if you agree).

I will send through WhatsApp (or email, if you do not have WhatsApp) the informed consent form for the participant to sign, indicating their agreement to participate in this stage of the research.

Final Thank You and Question about Consultation/Blood Collection:

On behalf of UFSC, we thank you for your participation and collaboration. We take this opportunity to verify if you are interested in taking part in the second stage of this research, which will be scheduled in the short term. This will consist of a nutritional care appointment designed for you, offering guidance on improving your diet for cancer prevention or your new cancer diagnosis. If you agree to participate, blood collection will also be done at this time to identify associated genetic tests, all with your authorization.

If not:

On behalf of UFSC, we thank you for your attention and wish you a good day.

In Case of Death:

We are very sorry for your loss. My name is (interviewer's name), I am a student at UFSC, and I am participating in a research project on nutrition and breast cancer. I contacted you regarding the participation of Mrs. (patient's name) in this project a few months ago. She was accompanied by nutritionists during her consultations, and she spoke about her diet.

We are currently collecting additional information over the phone to complement this research. We would like to know if you could participate by answering a few questions regarding Mrs. (patient's name), which will take only about 8 minutes.

If so:

1. **Before starting the questions**, I would like to emphasize that the information will remain confidential (identifying participants will not be used and will only be accessed after consent to participate in this research stage). Therefore, we would like to send you a Free and Informed Consent Form. Would you (sir/ma'am) agree to participate in this stage of the research? Would you (sir/ma'am) provide your phone number (WhatsApp) so we can send you the link to this form? [If you don't use WhatsApp, ask if you have an email.]
2. What is your full name, sir/ma'am?
3. Could you inform me when Mrs. (patient's name) passed away? (Preferably with the exact date.)
4. What was the cause of Mrs. (patient's name)'s death?
   On the death certificate, information such as what is called CID (ICD in English) is recorded. Would you be able to inform the code used in this statement? [Tell the respondent they can wait for the document if necessary; if the respondent prefers not to check it now, say they can wait for the next call to confirm and schedule a new contact calmly to retrieve this information.]
   (If there is a document, ask for the exact date of death and the ICD code; if this data isn't in the document, arrange to retrieve it later.)
5. After her breast cancer treatment, did Mrs. (patient's name) have any new case of cancer in the same breast or in another body part?
   [If yes, ask where in the body and confirm the location of the new cancer.]
6. If she had a recurrence: Could you inform me when she was diagnosed with this second tumor? If there is a document containing this information, could you share the date with us? [Say the participant can wait for a moment to retrieve the document; if the participant can't find it, say they can check later, and schedule another call calmly to confirm and retrieve the information.]
7. **[If recurrence occurred]** Did Mrs. (patient's name) undergo any treatment for this second tumor, such as surgery, chemotherapy, radiotherapy, or hormone therapy? If so, what treatments were performed?
   [If surgery was performed, also ask what type of surgery it was—for example: partial mastectomy, radical mastectomy (total), or oophorectomy.]
8. **[If recurrence occurred and treatment was performed]** In which hospital/healthcare institution did Mrs. (patient's name) undergo this/these treatment(s) for the second cancer?
9. **[If recurrence occurred and treatment was performed]** Could you inform me when this/these treatment(s) began and ended?
10. Did Mrs. (patient's name) have any other health issues, aside from cancer? Which ones?
11. Was she taking any continuous-use medication before her death? If so, which ones?

[If the participant needs time to retrieve some information]
In that case, I will call you (sir/ma'am) tomorrow to get the information contained in the documents about the diagnosis of the [first] cancer and [if recurrence occurred] the second cancer.

I will send you via WhatsApp [or email, if WhatsApp is not available] the Free and Informed Consent Form so you (sir/ma'am) can sign it, agreeing or not agreeing to participate in this stage of the research.

Final Gratitude Message:
On behalf of UFSC, we thank you for your participation and collaboration. Have a good day.

If not:
On behalf of UFSC, we thank you for your attention. Have a good day.

UFSC, Federal University of Santa Catarina.

Table S2. Unadjusted analysis of the clinical outcomes of breast cancer women according to specific components of the 2018 WCRF/AICR recommendations (1st tertile versus 2nd and 3rd tertiles), 2025.

**WCRF/AICR recommendation**	**Overall mortality (n = 24*)**		**Breast cancer-specific mortality (n = 15*)**		**Overall 10-year survival[a] (n = 84*)**		**Recurrence (n = 36*)**		**10-year recurrence[b] (n = 24*)**	
	HR (CI 95%)	Unadjusted analysis[#] (*p*)	HR (CI 95%)	Unadjusted analysis[#] (*p*)	HR (CI 95%)	Unadjusted analysis[#] (*p*)	HR (CI 95%)	Unadjusted analysis[#] (*p*)	HR (CI 95%)	Unadjusted analysis[#] (*p*)
**BMI**	1.05 (0.96-1.14)	0.266	0.94 (0.78-1.13)	0.525	0.98 (0.94-1.03)	0.564	1.00 (0.93-1.07)	0.905	1.05 (0.95-1.17)	0.300
**Waist circumference**	1.01 (0.97-1.04)	0.513	0.95 (0.89-1.03)	0.266	0.99 (0.98-1.01)	0.868	1.00 (0.98-1.03)	0.555	1.03 (0.99-1.06)	0.069
**Physical activity practice**	0.99 (0.99-1.00)	0.626	0.99 (0.99-1.00)	0.596	0.99 (0.99-1.00)	0.917	1.00 (0.99-1.00)	0.690	0.99 (0.99-1.00)	0.091
**Daily consumption of fruits and vegetables**	0.99 (0.99-1.00)	0.912	0.99 (0.99-1.00)	0.065	1.00 (0.99-1.00)	0.171	0.99 (0.99-1.00)	0.539	1.00 (0.99-1.00)	0.363
**Daily fiber intake**	0.98 (0.95-1.01)	0.426	0.93 (0.85-1.00)	0.075	1.00 (1.01-1.02)	**0.027**	0.98 (0.96-1.00)	0.240	1.02 (0.98-1.06)	0.203
**Consumption of ultra-processed foods**	10.41 (0.32-335.93)	0.186	32.7 (0.05-187.8)	0.282	0.43 (0.07-2.56)	0356	0.72 (0.02-20.11)	0.851	0.93 (0.01-430.08)	0.984
**Weekly consumption of red and processed meat**	0.99 (0.99-1.00)	0.967	1.00 (0.99-1.00)	0.963	0.99 (0.99-1.00)	0.514	0.99 (0.99-1.00)	0.412	0.99 (0.99-1.00)	0.063
**Daily consumption of sugary drinks**	1.01 (1.01-1.02)	**0.005**	1.00 (0.99-1.01)	0.375	1.00 (0.99-1.00)	0.129	1.00 (0.99-1.00)	0.156	1.00 (0.99-1.00)	0.816
**Daily alcohol consumption**	1.01 (0.92-1.10)	0.802	1.02 (0.89-1.15)	0.761	0.99 (0.96-1.03)	0.930	0.98 (0.95-1.02)	0.540	0.92 (0.79-1.08)	0.349

[#]Cox proportional hazards regression model. BMI, Body Mass Index; CI 95%, 95% confidence interval; HR, Hazard ratio; WCRF/AICR, World Cancer Research Fund/American Institute for Cancer Research. *Numbers of events for each outcome. [a]Overall 10-year survival refers to women who did not die within 10 years of the first diagnosis of breast cancer. [b]The term “10-year recurrence" refers to recurrence within 10 years of the primary diagnosis of breast cancer. **In bold are significant p-values.**

Table S3. Adjusted analysis of the clinical outcomes (overall mortality, breast cancer-specific mortality and overall 10-year survival) of breast cancer women according to specific components of the 2018 WCRF/AICR recommendations (1st tertile versus 2nd and 3rd tertiles), 2025.

WCRF/AICR recommendation	Overall mortality (n = 24*)				Breast cancer-specific mortality (n = 15*)				Overall 10-year survival[c] (n = 84*)			
	Hazard ratio (CI 95%)	Adjusted analysis (*p*)[a]	Hazard ratio (CI 95%)	Adjusted analysis (*p*)[b]	Hazard ratio (CI 95%)	Adjusted analysis (*p*)[a]	Hazard ratio (CI 95%)	Adjusted analysis (*p*)[b]	Hazard ratio (CI 95%)	Adjusted analysis (*p*)[a]	Hazard ratio (CI 95%)	Adjusted analysis (*p*)[b]
**BMI**	1.04 (0.90-1.21)	0.517	0.98 (0.89-1.07)	0.699	1.02 (0.70-1.50)	0.888	1.04 (0.92-1.17)	0.489	1.04 (0.92-1.18)	0.472	0.98 (0.94-1.03)	0.659
**Waist circumference**	1.01 (0.95-1.06)	0.652	0.99 (0.95-1.02)	0.641	0.94 (0.79-1.10)	0.474	1.02 (0.97-1.07)	0.389	0.99 (0.92-1.08)	0.976	0.99 (0.98-1.01)	0.875
**Physical activity practice**	0.99 (0.99-1.00)	0.778	0.99 (0.99-1.00)	0.914	0.98 (0.97-1.00)	0.153	0.99 (0.99-1.00)	0.705	0.98 (0.96-1.00)	0.082	1.00 (0.99-1.00)	0.680
**Daily consumption of fruits and vegetables**	1.00 (0.99-1.00)	0.326	0.99 (0.99-1.00)	0.409	0.97 (0.95-0.99)	**0.022***	0.99 (0.99-1.00)	0.283	0.99 (0.99-1.00)	0.886	1.00 (0.99-1.00)	0.238
**Daily fiber intake**	0.98 (0.94-1.03)	0.572	0.96 (0.93-1.00)	0.084	0.85 (0.73-1.00)	0.056	0.94 (0.88-1.00)	0.074	0.87 (0.74-1.03)	0.127	1.01 (1.01-1.02)	**0.022***
**Consumption of ultra-processed foods**	5.14 (0.07-372.6)	0.453	0.11 (0.01-10.51)	0.345	112.58 (0.01-2.97)	0.216	0.47 (0.01-124.4)	0.792	0.40 (0.01-162.78)	0.768	0.52 (0.08-3.27)	0.492
**Weekly consumption of red and processed meat**	1.00 (0.99-1.00)	0.701	1.00 (0.99-1.00)	0.500	1.00 (0.99-1.00)	0.887	1.00 (0.99-1.00)	0.887	1.00 (0.99-1.00)	0.229	0.99 (0.99-1.00)	0.089
**Daily consumption of sugary drinks**	1.01 (1.01-1.02)	**0.026***	1.00 (0.99-1.00)	0.825	1.01 (0.99-1.03)	0.121	1.00 (0.99-1.00)	0.244	1.00 (0.99-1.00)	0.866	1.00 (0.99-1.00)	0.090
**Daily alcohol consumption**	1.04 (0.91-1.19)	0.525	0.94 (0.87-1.01)	0.125	0.95 (0.65-1.38)	0.804	0.92 (0.81-1.04)	0.221	1.00 (0.86-1.17)	0.949	0.97 (0.94-1.01)	0.306

[a] Cox proportional hazards regression models adjusted for age, education, stage of first cancer, and family history of cancer. [b] Cox proportional hazards regression models adjusted for "a" and recurrence. [c]Overall 10-year survival refers to women who did not die within 10 years of the first diagnosis of breast cancer. *Numbers of events for each outcome. BMI, Body Mass Index; WCRF/AICR, World Cancer Research Fund/American Institute for Cancer Research. **In bold are significant p-values.**

Table S4. Adjusted analysis of the clinical outcomes (recurrence and 10-year recurrence) of breast cancer women according to specific components of the 2018 WCRF/AICR recommendations (1st tertile versus 2nd and 3rd tertiles), 2025.

WCRF/AICR recommendation	Recurrence (n = 36*)		10-year recurrence[a] (n = 24*)	
	HR (CI 95%)	Adjusted analysis (*p*)[b]	HR (CI 95%)	Adjusted analysis (*p*)[b]
**BMI**	0.99 (0.92-1.07)	0.970	1.05 (0.94-1.18)	0.344
**Waist circumference**	1.00 (0.98-1.03)	0.562	1.02 (0.98-1.07)	0.226
**Physical activity practice**	1.00 (0.99-1.00)	0.826	0.99 (0.98-1.00)	0.080
**Daily consumption of fruits and vegetables**	0.99 (0.99-1.00)	0.601	1.00 (0.99-1.00)	0.575
**Daily fiber intake**	0.98 (0.95-1.01)	0.324	1.02 (0.98-1.07)	0.247
**Consumption of ultra-processed foods**	0.63 (0.01-21.24)	0.799	0.11 (0.01-64.7)	0.506
**Weekly consumption of red and processed meat**	0.99 (0.99-1.00)	0.520	0.99 (0.99-1.00)	0.382
**Daily consumption of sugary drinks**	1.00 (0.99-1.00)	0.070	1.00 (0.99-1.00)	0.125
**Daily alcohol consumption**	0.98(0.93-1.02)	0.386	0.96 (0.80-1.15)	0.686

[a]The term "10-year recurrence" refers to recurrence within 10 years of the primary diagnosis of breast cancer. [b] Cox proportional hazards regression models adjusted for age, education, stage of first cancer and family history of cancer. *Numbers of events for each outcome. BMI, Body Mass Index; CI 95%, 95% confidence interval; HR, Hazard ratio; WCRF/AICR, World Cancer Research Fund/American Institute for Cancer Research.
